# Chaotic homes and school achievement: a twin study

**DOI:** 10.1111/j.1469-7610.2011.02421.x

**Published:** 2011-06-15

**Authors:** Ken B Hanscombe, Claire MA Haworth, Oliver SP Davis, Sara R Jaffee, Robert Plomin

**Affiliations:** King's College London, MRC Social, Genetic & Developmental Psychiatry Centre, Institute of PsychiatryLondon, UK

**Keywords:** Gene–environment correlation, household chaos, environmental confusion, home environment, school achievement, twin studies, behavioural genetics

## Abstract

**Background:**

Chaotic homes predict poor school performance. Given that it is known that genes affect both children's experience of household chaos and their school achievement, to what extent is the relationship between high levels of noise and environmental confusion in the home, and children's school performance, mediated by heritable child effects? This is the first study to explore the genetic and environmental pathways between household chaos and academic performance.

**Method:**

Children's perceptions of family chaos at ages 9 and 12 and their school performance at age 12 were assessed in more than 2,300 twin pairs. The use of child-specific measures in a multivariate genetic analysis made it possible to investigate the genetic and environmental origins of the covariation between children's experience of chaos in the home and their school achievement.

**Results:**

Children's experience of family chaos and their school achievement were significantly correlated in the expected negative direction (*r* = −.26). As expected, shared environmental factors explained a large proportion (63%) of the association. However, genetic factors accounted for a significant proportion (37%) of the association between children's experience of household chaos and their school performance.

**Conclusions:**

The association between chaotic homes and poor performance in school, previously assumed to be entirely environmental in origin, is in fact partly genetic. How children's home environment affects their academic achievement is not simply in the direction environment → child → outcome. Instead, genetic factors that influence children's experience of the disordered home environment also affect how well they do at school. The relationship between the child, their environment and their performance at school is complex: both genetic and environmental factors play a role.

Children who do better at school tend to come from homes that are quieter, more organized and have a predictable routine, regardless of socioeconomic status (Evans, 2006). Children living in the environmental confusion and unpredictability of high levels of family chaos (i.e. noise, disorder and human traffic) have lower expectations, lack of persistence and a tendency to withdraw from academic challenge (Brown & Low, 2008). The level of family chaos affects early reading skill, even after considering other home environmental factors relevant to children's mastery of reading (Johnson, Martin, Brooks-Gunn, & Petrill, 2008). It would be reasonable to conclude that home chaos has an environmental effect on school outcomes, but there is a potential confound – genes.

We know that school achievement is heritable; genes explain about half of the variation in academic ability (Kovas, Haworth, Dale, & Plomin, 2007; Petrill & Wilkerson, 2000). What about the home environment? With a genetically sensitive design such as the twin design, we can explore the genetic and environmental contributions to a particular ‘environment’. When twins are asked to rate the level of chaos in their home, identical twins who share all their genes are more similar in their experience than are nonidentical twins, suggesting that genes influence chaos (Hanscombe, Haworth, Davis, Jaffee, & Plomin, 2010). Genetic influence on an environmental measure, known as gene–environment correlation (Jaffee & Price, 2007; Kendler & Eaves, 1986; Plomin, DeFries, & Loehlin, 1977), means that the environment is not a passive event that just happens to us – we elicit reactions and construct the environment around us in part due to our genetic propensities.

Nevertheless, it seems reasonable to assume that the effect of home chaos on school performance is mediated environmentally, for example, by way of its effect on children's ability to complete their homework because of interruptions and distractions. However, it has been difficult to assess the origins of the association between home chaos and school performance because child-specific measures of chaos are needed to investigate this question. Given that children's perception of chaos in their home shows genetic influence, it is possible that the association is, in part, mediated genetically in the sense that common genes affect both chaos and achievement. Using a genetically sensitive design, it is possible to estimate the relative roles of genes and environments on the relationship between chaos and achievement. Knowing how nature and nurture work together in educationally relevant environments will inform the design of targeted interventions that could improve both child welfare and academic performance. In this study, we used the twin design to investigate the genetic and environmental contributions to the link between child reports of family chaos and their teacher-reported school achievement.

## Chaos is typically measured by parent reports

Parents’ reports of chaos in the home predict children's behaviour problems (Coldwell, Pike, & Dunn, 2006), lower cognitive test scores (Hart, Petrill, Deckard, & Thompson, 2007; Petrill, Pike, Price, & Plomin, 2004) and poor school performance (Brody & Flor, 1997). However, family-wide descriptions of the home environment provided by a parent cannot inform us about factors important for individual differences in the experiences of each child. That is, parent reports are not child-specific because they give just one account of the home for all the children living in it. This view of the home environment – that it is the same for all children living in it – is limited because it does not take into account the influence that each child exerts on their environment, including the genetic contribution to their experience through their behaviour. A child's environment has an impact on the child, but the child can also have an impact on their environment: there is a two-way relationship. Using measures of the experience of each individual within a genetically sensitive design has revealed that people's genes affect their experience (Kendler & Baker, 2007; Plomin & Bergeman, 1991). For example, aspects of the home environment (e.g. parental involvement and responsivity) measured on each child in the home were used to show that genetic factors explain about a quarter of the relationship between these characteristics of the home and standardized test-assessed achievement (Cleveland, Jacobson, Lipinski, & Rowe, 2000). For this reason, it is important to assess child-specific experiences of home chaos., to supplement the family-wide measures. This approach allows the investigation of genetic and environmental influences on environmental confusion and routine at home, and its association with outcomes such as school achievement.

Using child-specific measures we have shown that genetic factors do explain a significant proportion of individual differences in children's perceptions of chaos in the home between the ages of 9 and 12 years in the present sample (Hanscombe et al., 2010). Around 20% of the variation in experience of chaos is driven by heritable factors. What does it mean to say that an environment is heritable? Genetic influence on behaviours that affect *exposure* to, or experience of, the environment is called gene–environment (GE) correlation (Jaffee & Price, 2007; Kendler & Eaves, 1986; Plomin et al., 1977). There are three possible mechanisms: *passive* GE correlation happens because the environment children experience reflects their parents’ genetically influenced behaviour – children inherit both their parents’ genes and environment; *evocative* GE correlation is the result of people in the children's environment reacting to the children's genetically influenced behaviour or characteristics; *active* GE correlation arises when children directly seek out, select and modify their environment to suit their genetic propensities. The ‘environment’ is not something that simply happens to us. Instead, we seek environmental niches, modify our surroundings, select social interactions and engage other people in ways that are consistent with our genetic predispositions (Scarr & McCartney, 1983).

Given that both school achievement and home chaos show genetic influence, and that there is a correlation between them, we hypothesized that genetic factors would contribute to the association between chaotic homes and school achievement. We have measured school achievement at age 12; this age marks the transition to secondary school, the stage at which children are making choices about the subjects they will go on to study, as well as the age at which some children begin to drop out of school. Our aim was to assess the relative contribution of genetic and environmental factors to the association between chaos in the home and school achievement, using child-specific measures in a multivariate genetically sensitive twin design. We compared the resemblance of identical and nonidentical twins to find the genetic and environmental sources of covariation between chaos in the home and school achievement. As children rated chaos in their homes and teachers rated school achievement, we could rule out the confounding effects of having the same rater describe both environment and outcome.

## Methods

### Sample

The sample was drawn from the ongoing Twins Early Development Study, TEDS (Oliver & Plomin, 2007; Trouton, Spinath, & Plomin, 2002). TEDS is a population-based longitudinal study of over 10,000 pairs of twins born in England and Wales in 1994, 1995 and 1996. Informed consent was sought from the twins’ parents at each wave of assessment. The present study describes analyses of the twins’ perceptions of family chaos at ages 9 and 12, and their school achievement at age 12, measured on a subsample of 7,394 pairs in which we had data for at least one twin in a pair. Of these, 2,337 complete pairs had data on CHAOS at both 9 and 12 years; 3,040 complete pairs had data on school performance. Only the 1994 and 1995 birth cohorts were tested at age 9; all three birth cohorts were included in the 12-year wave of testing. In our analyses described next, we were able to make use of all the available data using full-information maximum likelihood procedures.

At both ages 9 and 12, the TEDS sample is representative of the UK general population. For example, UK census data for families with children indicate that 93% of children are white and 32% of mothers have at least one A-level (Advanced Level General Certificate of Education exams generally taken at age 18), and 49% of mothers and 89% of fathers are employed. For the entire TEDS sample, there are comparable percentages for ethnicity (92%), mother's education (35%) and mother's and father's employment status (43% and 92%, respectively). For the TEDS sample who participated at 9 years, the respective percentages are 94%, 41%, 46% and 93%; and at 12 years, the comparable percentages are 93%, 41%, 47% and 93%, respectively. Zygosity was assigned to the twins using a parent-rated instrument that yielded 95% accuracy when compared with zygosity established from DNA markers (Price et al., 2000); any uncertainty we followed up with DNA marker testing.

### Measures

#### Confusion, Hubbub and Order Scale

At 9 and 12 years the children's perceptions of chaos in the family home were assessed using a short version of the Confusion, Hubbub and Order Scale (CHAOS; Matheny, Wachs, Ludwig, & Phillips, 1995). The CHAOS scale has been widely used and has good psychometric properties; the original full-length inventory had high internal consistency (Cronbach's α = .79) and stability across a 12-month period (*r* = .74;Dumas et al. 2005). CHAOS was administered as part of a larger battery of measures in a booklet mailed to each of the twins. The short form of CHAOS assesses the level of routine, noise, and general environmental confusion with six items: ‘I have a regular bedtime routine’ (scoring reversed), ‘You can't hear yourself think in our home’, ‘It's a real zoo in our home’, ‘We are usually able to stay on top of things’ (scoring reversed), ‘There is usually a television turned on somewhere in our home’ and ‘The atmosphere in our house is calm’ (scoring reversed). The children rated the extent to which they agree: ‘not true’, ‘quite true’ or ‘very true’. At both ages 9 (Cronbach's α = .58) and 12 (Cronbachs's α = .57), a mean of the individual items was used as an overall score, with higher scores indicating greater chaos. Our internal consistency reliability is moderate and acceptable, although slightly lower than what others have found for parent ratings of the same short version in younger samples (e.g. .63, Petrill et al., 2004; .68, Hart et al., 2007). Child-reported CHAOS correlated .43 between ages 9 and 12. Parent-reported CHAOS when the children were 9 and 12 years of age correlated .53 and .55, respectively, with the corresponding child reports, supporting the validity of the child reports. Child-rated CHAOS at both ages was normally distributed.

#### School achievement

The assessment of school performance at age 12 was based on UK National Curriculum (NC) criteria [Qualifications and Curriculum Authority (QCA); http://curriculum.qca.org.uk/]. These criteria provide curriculum and assessment guidelines followed by all teachers in the UK state school system. The validity of teacher ratings has been demonstrated (Hoge & Colardaci, 1989); for example, in the current sample teacher assessments are highly correlated with standardized tests of reading and mathematics (Kovas et al., 2007). Teachers rated the children on each component of English, mathematics and science on a scale from 1 to 8, with an additional Level 9 for exceptional performance. This is a behaviourally anchored rating scale based on concrete targets; the QCA provides teachers with vignettes to standardize their assessments. As the children get older, different levels of the scale will come to represent the average expected performance. Children at age 12 have just begun Key Stage 3 of the UK NC, covering ages 11–14. At age 11, most pupils are expected to achieve Level 4 in the teacher assessments; at age 14, most pupils are expected to achieve Level 5. Children's performance is based on class work and homework, and takes account of written, practical and oral work.

**Figure 1:**
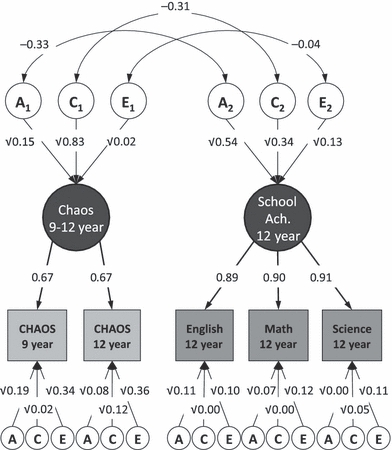
Common pathway model showing the genetic (A), shared (C) and nonshared (E) environmental relationship between latent factors representing child-reported CHAOS (Confusion, Hubbub and Order Scale) in the home between ages 9 and 12 years (CHAOS 9–12 yr) and teacher-reported school achievement at age 12 (School Ach. 12yr) for a sample population drawn from the Twins Early Development Study database

We calculated a mean score for each of the three subjects from teacher-rated NC levels of English (speaking and listening; reading; writing), mathematics (using and applying numbers; number and algebra; shape, space and measures; handling data) and science (scientific enquiry; life processes and living things; materials and their properties; physical processes) performance. English, mathematics and science were highly correlated at age 12 in the TEDS sample (see Table 1). The model we fitted to the data created a single latent factor of school achievement from the three subjects (see Figure 1).

**Table 1:** Phenotypic correlations between family chaos and achievement. CHAOS measures at ages 9 and 12, and English, mathematics and science at age 12 from a sample drawn from the TEDS database

	9 years	12 years			
		
	CHAOS	CHAOS	English	Mathematics	Science
9 years					
CHAOS	1				
	*N* = 3,123				
12 years					
CHAOS	.43	1			
	*N* = 2,484	*N* = 5,503			
English	−.16	−.18	1		
	*N* = 1,249	*N* = 3,205	*N* = 3,843		
Mathematics	−.17	−.16	.80	1	
	*N* = 1,208	*N* = 3,153	*N* = 3,738	*N* = 3,785	
Science	−.18	−.15	.82	.82	1
	*N* = 1,201	*N* = 3,143	*N* = 3,718	*N* = 3,721	*N* = 3,775

*N* indicates one randomly selected member from each twin pair (SPSS calculated). CHAOS, Confusion, Hubbub and Order Scale; TEDS, Twins Early Development Study.

### Statistical analysis

#### Classical twin design

Comparison of the resemblance between identical (monozygotic, MZ) twins and nonidentical (dizygotic, DZ) twins provides an estimation of the genetic and environmental contributions to variance within a trait and covariance between traits (Plomin, DeFries, McClearn, & McGuffin, 2008). Most of the human genome is identical from person to person, but a small proportion of it varies. If we just concentrate on the DNA that varies between humans, MZ twins are 100% identical. DZ twins, in contrast, are only 50% identical on average. So it follows that the extent to which MZ twins are more alike than DZ twins on any particular trait is a function of their greater genetic relatedness. Derived from quantitative genetic theory, the twin model partitions the variance of a trait, or the covariance between traits, into an additive genetic component (A), a shared (common) environmental component (C) and a nonshared environmental component (E; Evans, Gillespie, & Martin, 2002). The effect of the C component is to make reared together children similar on the trait of interest. Both C and A contribute to sibling similarity. E represents elements of the environment that uniquely affect reared-together siblings and therefore contributes to differences between twins. Any measurement error is included in the E term.

#### Twin model-fitting

We used structural equation modeling implemented in the program Mx (Neale, Boker, Xie, & Maes, 20602) to decompose the covariance structure of the relationship between child-reported CHAOS and teacher-reported school achievement. All available data were incorporated into the structural equation twin model-fitting procedure using full-information maximum likelihood. As illustrated in Figure 1, we fitted a common pathway model with two latent factors. One factor, ‘Chaos 9–12 yr’, represented children's ratings of chaos in the home between ages 9 and 12; the second factor, ‘School Ach. 12 yr’, indexed school achievement at age 12 from teacher ratings of English, math and science. Given that our aim was to assess the origin of the association between middle childhood chaos and school achievement, combining measures in a factor analysis provided a neat way to summarize the data to address this aim.

The common pathway model uses maximum likelihood factor analysis to derive latent factors. The variance of the latent factors is fixed to a value of 1 and partitioned into A, C and E components. Similarly, the residual variance of each of the measured traits is partitioned into A, C and E.

It is possible to assess model fit with a maximum likelihood estimate. The difference between twice the negative log-likelihood (Δ − 2ln*L*) of the data under a given model is distributed as chi-square (*χ*^2^), with degrees of freedom (*df*) equal to the difference in degrees of freedom between the two models. This can be used to test the relative fit of two models, although we report two other tests as well – Akaike's information criterion (AIC) and the Bayesian information criterion (BIC). AIC (AIC = Δ − 2ln*L* − 2 × Δ*df*;Akaike, 1987) imposes a penalty for each additional parameter and so provides a measure of model fit that favours parsimony, with lower AIC values indicating a better fit. The BIC [BIC = Δ − 2ln*L* − ln(*n*) × Δ*df*; Raftery, 1995] also favours parsimony, but unlike the AIC, this preference is independent of the sample size, and for this reason the BIC was the preferred index of fit for the current analyses. Lower BIC values indicate a better fit of the model to the data.

## Results

### Phenotypic analysis

The phenotypic correlation between child-reported CHAOS and teacher-reported school achievement is significant and negative [*r*_P_ = −.26, 95% confidence interval (CI) = −.30 to −.22], indicating that greater home chaos, as perceived by the child, are associated with worse performance in school. Table 1 shows the phenotypic correlations among chaos at ages 9 and 12, and English, mathematics and science at age 12.

Descriptive statistics and an analysis of variance by sex and zygosity for each of the five measures are presented in Table 2. The combined effect of zygosity and sex accounts for 1% or less of the variance for all five measures (*R*^2^ = .00–.01). For all subsequent analyses, the scores for men and women were combined.

**Table 2:** Means, standard deviations and analysis of variance by sex and zygosity for the sample population described in Table 1

		Male		Female		MZ		DZ		ANOVA				
						
Assessment	Measure	*M*	*SD*	*M*	*SD*	*M*	*SD*	*M*	*SD*	Sex	Zygosity	Sex × Zygosity	*R*^2^	*N*
9 years	CHAOS	0.77	0.39	0.71	0.38	0.74	0.39	0.75	0.38	<0.01	0.62	0.48	.01	3,123
12 years	CHAOS	0.69	0.34	0.64	0.34	0.67	0.34	0.67	0.34	<0.01	0.33	0.42	<.01	5,503
	English	4.28	0.96	4.43	0.88	4.32	0.91	4.34	0.95	<0.01	0.04	0.25	.01	3,843
	Mathematics	4.42	1.05	4.35	0.98	4.34	1.00	4.42	1.03	0.15	0.10	0.20	<.01	3,785
	Science	4.48	0.96	4.43	0.91	4.41	0.92	4.49	0.95	0.45	0.04	0.36	<.01	3,775

Assessment indicates age of assessment; *M*, mean; *SD*, standard deviation; MZ, monozygotic twins; DZ, dizygotic twins; sex indicates the *p*-value associated with sex effect on means; zyg. is the *p*-value associated with effect of zygosity on means; *R*^2^, proportion of the total variance explained by sex and zygosity; ANOVA, analysis of variance performed by using one randomly selected member of each twin pair; *N*, number of randomly selected individuals (one member of each twin pair) included in ANOVA analysis.

As similarity due to age and sex can contribute to phenotypic twin similarity and inflate estimates of C, the measures were corrected for the effects of age and sex, as is standard practice in the analysis of twin data (McGue & Bouchard, 1984). Age- and sex-corrected twin correlations by zygosity are shown in Table 3.

**Table 3:** Twin correlations and cross-twin correlations by zygosity, and ACE parameter estimates for chaos and achievement at ages 9 and 12 for the sample population described in Table 1

		Twin 2							
					
		9 years	12 years				Twin model estimates		
				
		1.	2.	3.	4.	5.	A	C	E
Twin 1									
9 years	1. CHAOS	**.66/.51**					.26 (.18–.34)	.39 (.32–.45)	.35 (.32–.38)
12 years	2. CHAOS	.46/.41	**.63/.56**				.15 (.09–.21)	.48 (.43–.53)	.37 (.34–.39)
	3. English	−.20/−.14	−.21/−.13	**.80/.53**			.56 (.50–.62)	.25 (.19–.31)	.19 (.17–.21)
	4. Mathematics	−.20/−.11	−.17/−.10	.68/.49	**.76/.53**		.49 (.43–.56)	.28 (.21–.34)	.23 (.21–.25)
	5. Science	−.23/−.14	−.19/−.14	.71/.49	.69/.51	**.76/.57**	.44 (.38–.50)	.34 (.28–.40)	.22 (.20–.24)

Along the diagonal, the values in bold are the within-trait cross-twin correlations (MZ/DZ); below the diagonal are the cross-trait cross-twin correlations (MZ/DZ). A, C and E indicate the proportion of phenotypic variance attributable to genetic, shared environmental and nonshared environmental factors, respectively (95% confidence intervals are shown within parentheses). MZ, monozygotic; DZ, dizygotic.

Along the diagonal in Table 3 are the within-trait twin correlations; below the diagonal are the cross-trait twin correlations. Doubling the difference between the MZ and DZ correlations within any trait gives an indication of the heritability. Within-trait across twin correlations suggest modest heritability for family chaos (average *h*^2^ = .22) and moderate heritability for teacher-rated NC achievement (average *h*^2^ = .53). Genetic model-fitting analyses described next provided a more comprehensive use of the data, and the possibility to fit a multivariate model with quantifying fit statistics.

### Genetic analyses

Variance components derived from univariate ACE models (see Table 3) suggest that the difference between child ratings of CHAOS at 9 and 12 years are not significant, as indicated by overlapping CIs. For example, the A component at age 9 is .26 (95% CI = .18–.34), and at age 12 is .15 (95% CI = .09–.21)). As a measure of long-term chaos, we combined the two measures into a single latent factor in our model. Figure 1 summarizes the common pathway ACE model fit to CHAOS at ages 9 and 12, and English, mathematics and science at age 12. The A, C and E variance components for the family chaos and school achievement factors are consistent with the univariate estimates in Table 3.

Compared with the saturated model (−2ln*L* = 88,604.36, *df* = 39,938), and a saturated model with means and variances constrained to be equal across twin and zygosity (Δ − 2ln*L* = 55.26, Δ*df* = 30, BIC = −133,696.10), the common pathway model did not provide a significantly worse account of the data (Δ − 2ln*L* = 177.28, Δ*df* = 99, BIC = −133,942.50). Considering model fit and parsimony, the common pathway model best explained the relationship between chaos and school achievement (more details in footnote to Table 4).

**Table 4:** Genetic and environmental correlations and bivariate estimates from the common pathway model

	Correlations			
	
Measure	*r*_A_	*r*_C_	*r*_E_	*r*_P_
CHAOS and achievement	−.33 (−1.00 to −.10)	−.31 (−0.43 to −0.19)	−.04 (−1.00 to 1.00)	−.26 (−0.30 to −0.22)

	Variance components of common factors			
		
	A	C	E	
				
CHAOS	.15 (0.02–0.28)	.83 (0.72–0.93)	.02 (0.00–0.07)	
Achievement	.54 (0.48–0.60)	.34 (0.28–0.39)	.13 (0.11–0.14)	

	Mediation of *r*_P_			
		
	*a*_*x*_*a*_*y*_*r*_A_/*r*_P_	*c*_*x*_*c*_*y*_*r*_C_/*r*_P_	*e*_*x*_*e*_*y*_*r*_E_/*r*_P_	
CHAOS and achievement	.37 (0.12–0.62)	.63 (0.41–0.84)	.01 (−0.07–0.08)	

*r*_A_, genetic correlation; *r*_C_, shared environmental correlation; *r*_E_, nonshared environmental correlation; *r*_P_, phenotypic correlation; *a*_*x*_*a*_*y*_*r*_A_/*r*_P_, proportion of phenotypic correlation mediated by genetic factors; *c*_*x*_*c*_*y*_*r*_C_/*r*_P_, proportion of the phenotypic correlation mediated by shared environmental factors; *e*_*x*_*e*_*y*_*r*_E_/*r*_P_, proportion of the phenotypic correlation mediated by nonshared environmental factors; 95% confidence intervals are shown within parentheses.

*Model fit:* 1. *Saturated*: −2LL = 88,604.36 (*df* = 39,938); 2. *Means/variances equal across twin and zygosity*: Δ − 2LL = 55.26 (Δ*df* = 30), *p* < .01, Akaike's information criterion (AIC) = −8,723.61, Bayesian information criterion (BIC) = −133,696.10; 3. *Common pathway*: Δ − 2LL = 177.28 (Δ*df* = 99), *p* < .01, AIC = 8,707.64, BIC = −133,942.50.

Table 4 shows the genetic and environmental correlations between the latent A, C and E components of variance. Both the shared environmental and the genetic correlation are significant and in the expected negative direction (*r*_C_ = −.31, 95% CI = −.43 to −.19; *r*_A_ = −.33, 95% CI = −1.00 to −.10). These correlations indicate that both shared environmental and genetic factors associated with household chaos will also be associated with school achievement.

The proportion of the phenotypic correlation explained by genetic and environmental factors –*bivariate* heritability and environmentality, respectively – is also shown in Table 4. The covariation between CHAOS and school achievement is largely shared environmental in origin (63%), however, genetic factors also explain a significant proportion (37%) of the phenotypic correlation. Nonshared environmental factors are unique to each trait and do not contribute to the association between experience of chaos and school achievement.

## Discussion

Consistent with previous studies using parental reports, we confirmed that children's experience of household chaos was associated with how well they performed in school. The more disorganized, noisy and confusing children perceived their homes to be, the poorer their performance in school. Environmental factors that make siblings more alike – shared environments – explained the largest part of the chaos–school achievement relationship. This might be expected considering chaos is after all a measure of the home environment, but noteworthy nonetheless given the recent rethinking about the effects of the shared environment (Burt, 2009). Remarkably, however, over a third of the association between children's perceptions of family chaos and their teacher-rated achievement was accounted for by common genetic factors.

### Environmental confusion at home predicts poor performance in school

Using a genetically sensitive design made it possible to characterize the influence of home environment on school achievement. By controlling for genetic effects, we have shown that about two-thirds of the association between chaos and school achievement is because of shared environmental factors. What could these shared experiences be? Obvious candidates are the elements of the scale itself, such as the items ‘I have a regular bedtime routine’, and ‘There is usually a television turned on somewhere in our home’. A previous study has found that the elements of the household chaos scale that tap order and routine (as opposed to noise) predict early reading skill (Johnson et al., 2008). This is supported by evidence that children living in unstable chaotic homes withdraw from academic challenge – an effect partially mediated by disrupted and inconsistent sleep patterns (Brown & Low, 2008). Poor ‘sleep hygiene’– irregular sleeping patterns including difficulty getting to sleep, staying asleep and excessive tiredness – is predictive of poor school performance (Bruni et al., 2006). Another characteristic of the chaotic home, immoderate television watching, both directly predict poor school performance and is significantly associated with disrupted sleep patterns (Li et al., 2007; Sharif & Sargent, 2010; Van Den Bulck, 2004). Of course, all these ‘environments’ are components of the heritable CHAOS scale, and are therefore likely themselves to be partly genetic in origin.

### Genetically driven experience: G → E correlation

The surprising finding here, however, is that the association between chaos and school achievement is not entirely environmental in origin. A common set of genetic factors explains a third of the association between the children's heritable experience of household chaos and their school achievement. But whose genes explain this relationship: the parents’ or the child's? If parents who create chaotic home environments also do not encourage schoolwork or take an interest in homework because of their genetic predisposition, the GE correlation between home and school is *passive*; parental genes bridge the children's experience of environmental confusion at home and their school performance. That is to say, children get their genes as well as their genetically influenced environment from their parents.

However, *passive* GE correlation on its own is only one step removed from a scenario in which the child, a blank slate, is entirely at the mercy of their nurture. Given that by the age of 12 we might expect that children are having some input into their routine at home and commitment to school, it seems likely that the genetic link between home and school is at least in part due to the child's genes: an *active* (or *reactive*) child-driven process. For example, if children are particularly uncooperative about going to bed, turning off the television or sitting down to meals, their parents may abandon attempts to impose structure on their environment. Similarly, the children's teachers may have to spend more time managing the children's behaviour than teaching them. Modifying the child's behaviour might allow parents to successfully implement regular routines and allow teachers to more effectively educate the child.

Another possibility is that some children become socially withdrawn as a way of filtering out the excess noise and confusion in chaotic homes (Evans, Rhee, Forbes, Mata Allen, & Lepore, 2000). Moreover, children in chaotic homes may be inappropriately extending this filtering to potentially beneficial social interactions and carrying it over to the classroom. If under the influence of genetic factors, a ‘tuning out’ strategy could explain the common genetic link between household chaos and school achievement. Notably, however, children's accounts of environmental confusion and disorder in the home predict school achievement even after accounting for problem behaviour and inattention in the present sample. The many potential behavioural mediators of the genetic link between chaotic homes and poor school performance are a rich area for exploration.

Finally, given that the present study measured perceptions of the environment by questionnaire, children's perceptions of the chaos in their homes could have been influenced by additional cognitive, affective and personality factors for genetic reasons. However, environments that are not measured by questionnaire are still found to be heritable (Plomin & Bergeman, 1991).

### Implications and future directions

This study highlights the importance of supplementing family-wide measures with individual-specific measures for the study of factors relevant to school achievement, and developmental outcomes in general. Child-specific measures within the genetically informative twin design provide a means to quantify the contribution of the child's (and their parents’) genes to their environment and its link to academic outcomes. To the extent that the link between chaotic homes and academic achievement is the result of shared experiences like unstructured television watching and irregular sleeping patterns, imposing structure will be beneficial. However, a common genetic contribution to the link between family chaos and low school performance suggests that additional targets for intervention may be found in as yet unidentified genetically driven behaviours of the child or their parents.

The present study focused on latent genetic and environmental factors linking family chaos and school achievement. However, underlying the genetic effect on experience of the chaotic home and its link to school achievement will be specific genetic variants. Isolating these variants and tracing out their effects, may tell us something about what behaviours or propensities underlie the heritable effect in children's experience of high levels of chaos in the home, and their poor performance in the classroom. If the common environmental component of the association between chaotic homes and school achievement represents a *causative* effect of home chaos on achievement – a possibility still to be tested – then imposing structure and order are obvious interventions. Future work to understand the shared environmental link between the experience of organization and routine at home and academic achievement will be informative about which routines and patterns are amenable to intervention. However, targeting behaviours like different children's perceptions and coping response when immersed in particular environments, may be a complementary strategy. Although the sample is representative of the UK population, the generalizability of the findings to populations in other countries, with different demographics, may be limited.

There are many potential background variables that could influence CHAOS and school achievement. These background variables are typically not twin-specific, but rather family-wide measures (e.g. socioeconomic status, SES) that could have an effect on the mean level of CHAOS and achievement, as well as a moderating effect on both measures and the link between them. Because ‘correction’ for obligatorily shared measures would have the same effect on both twins in a family, and our goal was to understand *individual differences*, we focused on the link between CHAOS as measured and school achievement in a genetically sensitive design; that is, on the origins of the covariation *within* pairs in context.

The environments we find ourselves in give opportunities to act out our genetic predispositions, to re-shape our surroundings and to select new environments and social interactions informed by our experience. We infuse the psychosocial environment of home with our particular blend of genetic preferences, and, as it turns out, some of the very same ingredients are evident in our school performance.

**Key points**

- Chaotic home environments, characterized by disorder, unpredictability and environmental confusion, are predictive of poor cognitive, behavioural and academic outcomes.
- But children play a role in selecting, modifying and shaping their home environment: a child's experience of family chaos is influenced by their genetic propensities.
- What this study shows is that common genes, as well as environments, link children's experience of the chaotic home and their school achievement.
- The implication of genetic effects on the chaos–achievement association is that genetically influenced behaviours (and perceptions) of the child and their parents are potential targets for intervention that will complement efforts to impose structure on the family home.
